# Long-Term Effects of Pneumococcal Conjugate Vaccine on Nasopharyngeal Carriage of *S. pneumoniae, S. aureus, H. influenzae* and *M. catarrhalis*

**DOI:** 10.1371/journal.pone.0039730

**Published:** 2012-06-25

**Authors:** Judith Spijkerman, Sabine M. P. J. Prevaes, Elske J. M. van Gils, Reinier H. Veenhoven, Jacob P. Bruin, Debby Bogaert, Alienke J. Wijmenga-Monsuur, Germie P. J. M. van den Dobbelsteen, Elisabeth A. M. Sanders

**Affiliations:** 1 Department of Pediatric Immunology and Infectious Diseases, Wilhelmina Children's Hospital, University Medical Center, Utrecht, The Netherlands; 2 Research Center Linnaeus Institute, Spaarne Hospital, Hoofddorp, The Netherlands; 3 Regional Laboratory of Public Health, Haarlem, The Netherlands; 4 Vaccinology Unit, National Institute for Public Health and the Environment, Bilthoven, The Netherlands; Instituto Butantan, Brazil

## Abstract

**Background:**

Shifts in pneumococcal serotypes following introduction of 7-valent pneumococcal conjugate vaccine (PCV-7) may alter the presence of other bacterial pathogens co-inhabiting the same nasopharyngeal niche.

**Methodology/Principal Findings:**

Nasopharyngeal prevalence rates of *S. pneumoniae, S. aureus, H. influenzae* and *M. catarrhalis* were investigated before, 3 and 4.5 years after introduction of PCV-7 in the national immunisation program in children at 11 and 24 months of age, and parents of 24-month-old children (n≈330/group) using conventional culture methods. Despite a virtual disappearance of PCV-7 serotypes over time, similar overall pneumococcal rates were observed in all age groups, except for a significant reduction in the 11-month-old group (adjusted Odds Ratio after 4.5 years 0.48, 95% Confidence Interval 0.34–0.67). Before, 3 and 4.5 years after PCV-7 implementation, prevalence rates of *S. aureus* were 5%, 9% and 14% at 11 months of age (3.59, 1.90–6.79) and 20%, 32% and 34% in parents (1.96, 1.36–2.83), but remained similar at 24 months of age, respectively. Prevalence rates of *H. influenzae* were 46%, 65% and 65% at 11 months (2.22, 1.58–3.13), 52%, 73% and 76% at 24 months of age (2.68, 1.88–3.82) and 23%, 30% and 40% in parents (2.26, 1.58–3.33), respectively. No consistent changes in *M. catarrhalis* carriage rates were observed over time.

**Conclusions/Significance:**

In addition to large shifts in pneumococcal serotypes, persistently higher nasopharyngeal prevalence rates of *S. aureus* and *H. influenzae* were observed among young children and their parents after PCV-7 implementation. These findings may have implications for disease incidence and antibiotic treatment in the post-PCV era.

## Introduction

The 7-valent pneumococcal conjugate vaccine (PCV-7; Prevenar™, Pfizer) prevents nasopharyngeal acquisition of 7 paediatric serotypes in children and transmission to others [1], [2]. This results in protection against PCV-7 serotype disease in all age groups [3]–[5]. The vacant nasopharyngeal niche in PCV-7 vaccinated children is immediately occupied by non-PCV-7 serotype pneumococci either due to true replacement, unmasking, or capsular switch, resulting in similar overall pneumococcal carriage rates [1].

Previous reports have demonstrated potential associations between pneumococcal presence in the nasopharynx, in particular vaccine serotypes, and bacteria like *H. influenzae, M. catarrhalis* or *S. aureus* co-inhabiting the same ecological niche [6]–[8]. This has raised the concern that shifts in pneumococcal serotypes after PCV-7 vaccination may facilitate the involvement of other major contributors to respiratory, as demonstrated in three otitis media efficacy trials [9]–[11], or invasive disease in childhood [12]–[14]. A temporary increase in *S. aureus* nasopharyngeal carriage at one year of age after PCV-7 vaccinations at 2, 4 and 11 months was previously reported in a randomized controlled trial [15]. Several years of nationwide PCV-7 vaccine pressure might enhance shifts in the bacterial ecosystem in infants. Young children are an important reservoir and major source of transmission of bacteria to the whole community [14], [16], [17]. Therefore, the aim of this study was to investigate the long-term effects of PCV-7 on nasopharyngeal carriage of *S. pneumoniae*, *S. aureus, H. influenzae* and *M. catarrhalis* in healthy PCV-7 vaccinated children and their unvaccinated parents in the Netherlands.

## Methods

### Ethics statement

Both cross-sectional studies were approved by an acknowledged independent ethics committee from the Netherlands (CCMO; Centrale Commissie Mensgebonden Onderzoek, available at: http://www.ccmo-omline.nl). Parents of children living in the western part of the Netherlands were provided with written information about the study and asked to participate. Written informed consent was obtained from all parents and the procedures followed were in accordance with European Statements for Good Clinical Practice and the Declaration of Helsinki of the World Medical Association.

### Study design

PCV-7 was implemented in the national immunization program (NIP) for all infants born after 31 March 2006 at 2, 3, 4 and 11 months of age without a catch-up campaign for older children. Traditionally, PCV-7 vaccine uptake reached almost 95% [18]. Two subsequent cross-sectional carriage studies of similar design were conducted to evaluate nasopharyngeal bacterial carriage after introduction of PCV-7 in the national immunization program (NIP): 1) 3 years post-PCV-7 (February through July 2009) and 2) 4.5 years post-PCV-7 (September 2010 through January 2011). In both cross-sectional carriage studies, nasopharyngeal bacterial carriage was evaluated by means of home visits in two age-cohorts of healthy children vaccinated according to the Dutch NIP: 1) 11-month-old children after 3 primary doses but who had not yet, or had within the last week, obtained the booster dose at 11 months, and 2) 24-month-old children after four doses. Additionally, nasopharyngeal samples were collected from one of the parents of each 24-month-old child. Exclusion criteria were known or suspected immunodeficiency, craniofacial or chromosomal abnormalities, coagulation disorders and use of anticoagulant medication. Data of the vaccinated children cohorts and their parents were compared with data from PCV-7 unvaccinated children at the age of 12 and 24 months and parents of 24-month-old children derived from a previous, randomized controlled trial that had started in the Netherlands well before national PCV7 implementation for infants (NCT00189020) [19]. In this trial, children had been included at the age of 6 weeks between July 2005 and February 2006 and were followed-up until 24 months of age.

### Procedures

Transnasal nasopharyngeal samples were obtained from children and parents by trained study personnel with a nylon flocked flexible sterile Copan E-swab according to World Health Organization standard procedures [20]. From parents, an additional transoral nasopharyngeal sample was collected with a rigid sterile swab under direct observation of the posterior pharynx, as the pneumococcal yield is known to be higher in adults when taking both samples [21]. After sampling, all swabs were directly inoculated in liquid Amies medium and plated within 24 hours. All the swabs of the three studies were processed by same laboratory and cultured for presence of *S. pneumoniae, S. aureus, H. influenzae* and *M. catarrhalis* according to standard bacteriological procedures for conventional culture. One pneumococcal colony per plate was subcultured and serotyped by Quellung reaction using type-specific antisera from the Statens Serum Institute (Copenhagen, Denmark). In a post-hoc analysis, the presence of methicillin resistance was determined in a random sample of 30 (65%) of the *S. aureus* strains obtained from children at 11 months of age 4.5 years after PCV-7 implementation by using disk diffusion method for cefoxitine and by detection of the mecA gene [22].

### Covariates

Parents of the participating children completed a brief survey on the following possible predictors of nasopharyngeal bacterial carriage: age, sex, season of sampling, antibiotic use within one month prior to sampling, symptoms of a respiratory tract infection and/or acute otitis media during sampling, presence of siblings in the household, day care attendance of the participating child, passive smoke exposure indoors, active smoking of the participating parent.

### Statistical analyses

This study was designed to demonstrate changes in pneumococcal carriage after PCV-7 implementation requiring 330 persons per group as described previously (4). Assessment of the effects of PCV-7 on carriage of *H. influenzae, M. catarrhalis* and *S. aureus* were designated secondary outcomes. Given estimated prevalence rates of 49%, 64% and 5% for *H. influenzae, M. catarrhalis* and *S. aureus* in children based on previous reports [13], [20], a sample size of 330 could detect with 80% power and a 2-sided α of 0.05 an increase of 10%, 9% and 6%, respectively. Differences in prevalence rates were tested using 2-sided χ2 or Fisher's exact test, where appropriate. P-values <0.05 were considered significant. Multivariate analysis with backward LR binary logistic regression modelling was used to obtain adjusted estimates of the association between the outcomes and intervention as given by adjusted odds ratio's (aOR) and their corresponding 95% confidence intervals (95% CI). All measured possible confounders were entered in this model and removed when p-value was >0.10. To obtain the aOR for the intervention effect in case the intervention variable was not included in the final model (i.e. p-value >0.10), this variable combined with all remaining variables of the final backward LR model were entered in a binary logistic regression model without selection (SPSS version 17.0).

## Results

Baseline characteristics of children and their parents before, 3 and 4.5 years after implementation of PCV-7 in the NIP are shown in Table 1. The most noticeable difference among all three time-points is the season of sampling.

**Table 1:** Characteristics of children and their parents before, 3 and 4.5 years after implementation of PCV-7 in the Netherlands.

		11 months			24 months		
		Pre-PCV7	3yr Post	4.5yr Post	Pre-PCV7	3yr Post	4.5yr Post
		no. (%)	no. (%)	no. (%)	no. (%)	no. (%)	no. (%)
**Children**		n = 319	n = 329	n = 330	n = 321	n = 330	n = 330
Male sex		156 (49)	181 (55)	173 (52)	155 (48)	187 (57)	171 (52)
Mean age in months (SD)		12.0 (0.3)	10.9 (0.3)	10.7 (0.4)	24.2 (0.6)	24.0 (0.3)	23.8 (0.5)
Presence of siblings <5yr		126 (40)	84 (26)	145 (44)	127 (40)	135 (41)	154 (47)
Day care attendance^a^		208 (65)	226 (69)	222 (67)	224 (70)	233 (71)	259 (79)
Passive smoke exposure^b^		21 (7)	5 (2)	9 (3)	26 (8)	16 (5)	12 (4)
Signs of URTI and/or OMA^c^		95 (30)	95 (29)	112 (34)	82 (26)	69 (21)	114 (35)
Antimicrobial drug use^d^		20 (6)	24 (7)	27 (8)	10 (3)	23 (7)	15 (5)
Period of sampling:							
	October-March	149 (47)	82 (25)	274 (83)	156 (48)	299 (91)	299 (91)
	April-September	170 (53)	247 (75)	56 (17)	166 (52)	31 (9)	31 (9)
		**Pre-PCV7**	**3yr Post**	**4.5yr Post**			
**Parents**		n = 296	n = 324	n = 326			
Male sex		51 (17)	53 (16)	58 (18)			
Mean age in years (SD)		34.7 (4.9)	35.1 (4.4)	35.3 (4.5)			
Active smoking		40 (14)	34 (11)	41 (13)			
Antimicrobial drug use^d^		9 (3)	20 (6)	16 (5)			

Note. PCV-7; 7-valent pneumococcal conjugate vaccine. SD; standard deviation. ^a^Defined as more than 4 hours per week with at least 1 child from a different household. ^b^Defined as passive tobacco smoke exposure indoors at least ≥1 cigar or cigarette during ≥5 days/week. ^c^The presence of symptoms of an upper respiratory tract infection (URTI) and/or acute otitis media (OMA) as defined by evaluation of parents. ^d^Defined as use of oral or intravenous antibiotics within 1 month before sample was taken.

### Carriage of *S. pneumoniae*

Three years after PCV-7 implementation (post-PCV7), a nearly complete eradication of PCV-7 serotype carriage in children and adults was demonstrated, resulting in significant declines in overall pneumococcal carriage at 11 and 24 months of age and similar carriage rates in parents [2]. After 4.5 years, PCV-7-serotypes as well as cross-reactive serotype 6A were found only incidentally in all age groups (Table S1). Compared to pre-PCV7, overall pneumococcal prevalence rates had remained significantly lower at 11 months of age after 4.5 years (adjusted Odds Ratio (aOR) 0.48, 95% Confidence Interval 0.34–0.67), but had risen to similar levels at 24 months of age and in parents (Table 2). Serotype 19A became the predominant colonizer in all age groups, followed by serotypes 6C, 15 B/C and 11A (Table S1).

**Table 2:** Frequencies of nasopharyngeal carriage and (adjusted) odds ratios of *S. pneumoniae, S. aureus, H. influenzae* and *M. catarrhalis* in children and parents before, 3 and 4.5 years after PCV-7 implementation.

	Pre-PCV7	3yr Post	4.5yr Post	3yr Post vs. Pre-PCV7		4.5yr Post vs. Pre PCV7		4.5yr Post vs. 3yr Post PCV7	
	no. (%)	no. (%)	no. (%)	OR (95% CI)	aOR (95% CI)	OR (95% CI)	aOR (95% CI)	OR (95% CI)	aOR (95% CI)
**11 months^a^:**	n = 319	n = 329	n = 330						
*S. pneumoniae*	214 (67)	154 (47)	173 (52)	0.43 (0.31–0.59)	0.43 (0.30–0.60)	0.54 (0.39–0.74)	0.48 (0.34–0.67)	1.25 (0.92–1.70)	1.10 (0.79–1.53)
PCV-7-serotypes	122 (38)	25 (8)	10 (3)	0.13 (0.08–0.21)	0.14 (0.09–0.23)	0.05 (0.03–0.10)	0.04 (0.02–0.08)	0.38 (0.18–0.80)	0.32 (0.15–0.69)
Non-PCV-7- serotypes	92 (29)	129 (39)	166 (50)	1.59 (1.15–2.21)	1.75 (1.24–2.46)	2.50 (1.81–3.46)	2.59 (1.85–3.62)	1.57 (1.15–2.14)	1.47 (1.05–2.05)
*S. aureus*	16 (5)	31 (9)	46 (14)	1.97 (1.06–3.68)	1.97 (1.04–3.72)	3.07 (1.70–5.54)	3.59 (1.90–6.79)	1.56 (0.96–2.53)	1.88 (1.14–3.13)
*H. influenzae*	146 (46)	212 (64)	213 (65)	2.15 (1.57–2.94)	2.69 (1.90–3.80)	2.16 (1.57–2.96)	2.22 (1.58–3.13)	1.01 (0.73–1.38)	0.83 (0.59–1.17)
*M. catarrhalis*	218 (68)	210 (64)	251 (76)	0.82 (0.59–1.13)	0.89 (0.62–1.27)	1.47 (1.04–2.08)	1.25 (0.85–1.83)	1.80 (1.28–2.53)	1.82 (1.27–2.61)
**24 months^a^:**	n = 321	n = 330	n = 330						
*S. pneumoniae*	211 (66)	162 (49)	211 (64)	0.50 (0.37–0.69)	0.50 (0.36–0.70)	0.92 (0.67–1.28)	0.81 (0.58–1.13)	1.84 (1.35–2.51)	1.73 (1.26–2.38)
PCV-7-serotypes	114 (36)	14 (4)	11 (3)	0.08 (0.05–0.14)	0.08 (0.05–0.15)	0.06 (0.03–0.12)	0.06 (0.03–0.12)	0.78 (0.35–1.74)	0.76 (0.34–1.70)
Non-PCV-7- serotypes	97 (30)	148 (45)	200 (61)	1.88 (1.36–2.59)	1.99 (1.43–2.77)	3.55 (2.57–4.92)	3.35 (2.40–4.67)	1.89 (1.39–2.58)	1.78 (1.30–2.44)
*S. aureus*	18 (6)	25 (8)	24 (7)	1.38 (0.74–2.58)	1.39 (0.74–2.63)	1.32 (0.70–2.48)	1.61 (0.84–3.07)	0.96 (0.54–1.71)	1.09 (0.60–1.97)
*H. influenzae*	168 (52)	240 (73)	252 (76)	2.43 (1.75–3.37)	2.70 (1.91–3.82)	2.94 (2.10–4.12)	2.68 (1.88–3.82)	1.21 (0.85–1.72)	0.99 (0.68–1.44)
*M. catarrhalis*	189 (59)	199 (60)	264 (80)	1.06 (0.78–1.45)	1.37 (0.97–1.94)	2.84 (2.00–4.03)	1.75 (1.18–2.61)	2.67 (1.89–3.79)	1.37 (0.85–2.21)
**Parents^b^:**	n = 296	n = 324	n = 326						
*S. pneumoniae*	50 (17)	51 (16)	66 (20)	0.92 (0.60–1.41)	0.91 (0.59–1.40)	1.25 (0.83–1.88)	1.22 (0.81–1.84)	1.36 (0.91–2.03)	1.33 (0.89–2.00)
PCV-7-serotypes	25 (8)	2 (1)	7 (2)	0.07 (0.02–0.29)	0.07 (0.02–0.29)	0.24 (0.10–0.56)	0.24 (0.10–0.55)	3.53 (0.73–17.14)	4.09 (0.83–20.05)
Non-PCV-7- serotypes	25 (8)	49 (15)	59 (18)	1.93 (1.16–3.22)	1.93 (1.16–3.23)	2.50 (1.52–4.10)	2.43 (1.48–4.00)	1.29 (0.86–1.95)	1.25 (0.82–1.89)
*S. aureus*	60 (20)	104 (32)	111 (34)	1.86 (1.29–2.68)	1.85 (1.36–2.83)	2.03 (1.41–2.92)	1.96 (1.36–2.83)	1.09 (0.79–1.51)	1.07 (0.77–1.49)
*H. influenzae*	67 (23)	96 (30)	130 (40)	1.44 (1.00–2.07)	1.49 (1.03–2.14)	2.27 (1.60–3.22)	2.26 (1.58–3.33)	1.58 (1.14–2.18)	1.52 (1.10-2.11)
*M. catarrhalis*	28 (10)	43 (13)	71 (22)	1.47 (0.88–2.43)	1.44 (0.87–2.39)	2.67 (1.67–4.26)	2.54 (1.59–4.08)	1.82 (1.20–2.76)	1.77 (1.17–2.69)

Note. PCV-7; all serotypes included in 7-valent pneumococcal conjugate vaccine. Non-PCV-7; all other serotypes not included in 7-valent pneumococcal conjugate vaccine. OR; odds ratio.CI; confidence interval. aOR; adjusted odds ratio. All (a)OR-values are based on comparison with cohort as indicated. ^a^In children, OR-values were adjusted by multivariate analysis for sex, months of sampling, presence of siblings <5yr in the household, day care attendance, antibiotic use, passive smoke exposure and the presence of URTI and/or OMA using binary logistic regression with backward LR. ^b^In parents, OR-values were adjusted by multivariate analysis for sex, months of sampling, antibiotic use, active smoking, presence of siblings <5yr other than participating child and day care attendance of their participating child.

### Carriage of *S. aureus*

The prevalence of *S. aureus* was significantly higher in children at 11 months of age (aOR 1.97, 1.04–3.72) and in parents (1.85, 1.36–2.83) 3 years post-PCV7 compared to pre-PCV7. After 4.5 years, a further increase in the prevalence of *S. aureus* was observed at 11 months of age (2.26, 1.58–3.33) compared to pre-PCV7 and results persisted in parents. No changes in *S. aureus* carriage were observed in children at 24 months of age after 3 and 4.5 years post-PCV7 (Table 2).No methicillin resistance was detected in tested isolates (n = 30) obtained from 11-month-old children 4.5 years post-PCV-7.

### Carriage of *H. influenzae*

The prevalence of *H. influenzae* was significantly higher in children at 11 months (aOR 2.69, 1.90–3.80), 24 months of age (2.70, 1.91–3.82) and parents (1.49, 1.03–2.14) after 3 years of PCV-7 implementation compared to pre-PCV7. After 4.5 years, higher levels persisted in children, and a further increase in prevalence of *H. influenzae* was observed in parents (2.26, 1.58–3.33) compared to pre-PCV7 (Table 2).

### Carriage of *M. catarrhalis*

The prevalence of *M. catarrhalis* remained unchanged in children and parents 3 years post-PCV7 as compared to pre-PCV7. After 4.5 years, the prevalence of *M. catarrhalis* remained similar at 11 months of age, but was significantly higher in children at 24 months of age (aOR 1.75, 1.18–2.61) and in parents (2.54, 1.59–4.08) compared to pre-PCV7 (Table 2).

## Discussion

This is the first study that describes the long-term follow-up of nasopharyngeal carriage of *S. aureus, H. influenzae* and *M. catarrhalis* next to *S. pneumoniae* after introduction of PCV-7 in the NIP. In addition to the virtual elimination of PCV-7 serotypes, we demonstrated significantly higher *S. aureus* carriage rates in children by the end of the first year of life and in the parents group. For *H. influenzae*, significantly higher prevalence rates were detected among children at both 11 and 24 months of age and in parents. Shifts in nasopharyngeal carriage profiles of these bacteria may affect disease incidence due to these pathogens in PCV-7 vaccinated children as well as in the population. Moreover, increased nasopharyngeal carriage may lead to increased exposure of strains to antibiotics, which may subsequently lead to selection of resistant ones [23].

The results on *S. aureus* carriage are consistent with a previous randomized controlled trial that demonstrated a doubled rate of *S. aureus* carriage in healthy children at 12 months of age after 2+1 doses of PCV-7 and in parents as compared to unvaccinated controls [15]. Similar to our present study, in this trial the impact of PCV-7 on *S. aureus* carriage in children was limited to young children around 12 months of age and no longer present at 18 or 24 months of age. This indicates an effect on *S. aureus* carriage when carriage dynamics are at their peak, around PCV-7 vaccinations in infancy, and disappearing later after non-PCV-7 serotypes have filled in the gap or by age-dependent maturation of the immune system in the second year of life. The impact of pneumococcal vaccinations on *S. aureus* presence is also in line with a randomized controlled trial where an increased rate of *S. aureus* culture–positive acute otitis media (AOM) was demonstrated shortly after pneumococcal vaccinations in children suffering from recurrent AOM, who were PCV-7-vaccinated followed by the 23-valent polysaccharide vaccine [11].

In support of these findings, several studies from other sites have also reported a negative interaction between *S. pneumoniae*, in particular PCV-7-serotypes, and *S. aureus* carriage in healthy children [6]–[8], [24] but also during severe pneumonia illness in HIV-negative children [25]. In contrast to these studies, two observational studies performed after introduction of PCV-7, one in children with acute otitis media and one in primary-care visiting children, did not yield an effect on *S. aureus* nasopharyngeal carriage [26], [27]. However, these studies included children from various age groups with different intervals between pneumococcal vaccinations and the sampling moment, and may not have detected the temporal impact of PCV-7 on *S. aureus* carriage around vaccinations in infancy. Furthermore, no comparison to a baseline level of *S. aureus* carriage before PCV-7 implementation could be made and several methodological concerns were raised [28].

One possible mechanism of interaction between vaccine serotype pneumococci and *S. aureus* is the presence of pneumococcal pilus predicting the absence of *S. aureus* carriage in pneumococcal carriers [29]. Host immunological factors may also play a role, as the negative interaction between *S. pneumoniae* and *S. aureus* is not reported in HIV-positive children [25]. No negative interaction between *S. pneumoniae* and *S. aureus* has been identified in adults. Although we cannot discriminate the route of intra-familial transmission in this cross-sectional study, we therefore hypothesize that vaccinated children induce more transmission of *S. aureus* to parents who become colonized for longer periods. Persistent carriage, as defined by continuous colonisation by the same strain of *S. aureus* in time, is a common phenomenon in adults but rarely detected in infants [8], [30].

The observed increase in nasopharyngeal carriage of *S. aureus* is worrisome as this is a known risk factor for infection [14], [31], and especially young children in first year of life are vulnerable for *S. aureus* disease [32]. In the last decade, only few population-based studies have been published on invasive *S. aureus* disease (ISD) after the introduction of PCV-7. Of those, two large studies from the USA reported a significant absolute increase in ISD, which could solely be attributed to an increase in MRSA [33], [34] and no other countries have reported substantial increases so far. Based on our results, long-term follow-up of staphylococcal carriage and ISD surveillance, especially after complete eradication of pneumococcal vaccine serotypes and following implementation of broader-coverage pneumococcal conjugate vaccines in the population, needs to be implemented.

The long-term impact of PCV-7 implementation on nasopharyngeal carriage of *H. influenzae* and *M. catarrhalis* was also investigated. Two Finnish otitis media trials investigating a CRM197- or a OMPC-protein based PCV-7 in healthy infants noted an increase in either *H. influenzae* or *M. catarrhalis* in middle-ear fluid during episodes of AOM, respectively [9], [10]. Post-marketing observational studies in the USA reported relative increases in both *H. influenzae* and *M. catarrhalis* causing AOM in children after PCV-7 implementation [35], [36].

Relatively high nasopharyngeal carriage rates of *H. influenzae* and *M. catarrhalis* were already observed before PCV-7 introduction in the Netherlands [37]. Nevertheless, we observed significant increases in *H. influenzae* carriage in children of both age groups as well in their parents both 3 and 4.5 years after introduction of PCV-7. Although no consistent changes in nasopharyngeal carriage of *H. influenzae* were observed by conventional culture techniques in a previous randomized controlled trial evaluating reduced-dose schedules of PCV-7 [37], a trend towards increased presence and abundance of *Haemophilus* species by molecular techniques was observed in PCV-7 vaccinated children compared with PCV-7 unvaccinated controls after 2+1 doses of PCV-7 (unpublished data). We therefore hypothesize that implementation of a full-dose schedule with 4 doses of PCV-7, together with continuing nationwide PCV-7 vaccine pressure, may have facilitated increased *H. influenzae* carriage in our study. In this study, we have not typed the recovered *H. influenzae* isolates. However, between 2008 and 2010, during the same period as the current study, we conducted a large randomized controlled trial in the same area in the Netherlands in which 780 infants were vaccinated with either CRM197-conjugated PCV-7 or Protein-D-conjugated PCV-10 with carriage of *H. influenzae* as endpoint. Similar to the present study, 65% of all cultured swabs were positive for *H. influenzae* based on conventional culture methods. After discrimination from *H. haemolyticus* by PCR, 92% were confirmed as *H. influenzae*. Of these, 8% were encapsulated strains; most frequently type a (5%) and f (1%) (unpublished data).

As for *M. catarrhalis,* no consistent changes in carriage after PCV-7 implementation were found, except for an increase in carriage in 24-month-old children and their parents 4.5 years post-PCV7. These results are in line with the randomized controlled trial which found no changes in *M. catarrhalis* carriage [37]. Again, bacterial interference, resource availability and host immunological factors may all play a role in the co-existence or competition between species in the nasopharynx, but the exact mechanisms of interactions are not yet fully elucidated [38].

With respect to the pneumococcal carriage surveillance study 4.5 years after PCV-7 introduction, we found that PCV-7-serotype pneumococci and cross-reactive serotype 6A were virtually eliminated among children and their parents. Simultaneously, non-PCV-7-serotypes increased significantly and showed higher diversity. Consistent with reports from other countries, 19A became the most predominant colonizing serotype in all investigated age groups in the Netherlands, followed by 6C, 15B/C and 11A [39], [40]. Also in IPD, serotype 19A has increased significantly to become the fourth most frequently isolated serotype in the Netherlands after 7F, 1 and 8. Although frequently carried serotypes 6C, 15B/C and 11A are now also more frequently found in IPD, their total number of cases still remained low (unpublished data). However, due to the adaptive nature of the pneumococcus [41], long-term nasopharyngeal carriage and IPD surveillance in all age groups remain essential to evaluate health and economic impact in the future.

A limitation of the current observational study design is that we documented associations and no causalities between the introduction of PCV-7 and changes in nasopharyngeal bacterial carriage patterns. However, after correction for differences in measured potential confounders by using multivariate analysis, our results remained robust. Nonetheless, the impact of other unmeasured determinants such as the presence of viruses or climatologic factors cannot be excluded. Also, our results for *S. aureus* may have been underestimated due to sampling the nasopharyngeal niche rather than sampling the anterior nares, which is assumed the ecological site for *S. aureus* [14].

Strengths of our study are the consistency of our data measured in large cohorts at two sequential time points after PCV-7 implementation and compared to a historical baseline. Importantly, similar standard operating procedures were followed in the same laboratory for all three studies. Furthermore, we evaluated the impact of PCV-7 not only in vaccinated infants but also in unvaccinated adult contacts in a country with PCV-7 uptake of almost 95% in the NIP [18]. Finally, the studies were performed in a country with low antibiotic consumption and resistance rates and therefore unlikely to be confounded by antibiotic resistant clones [42].

In conclusion, next to large shifts in pneumococcal serotypes, we observed persistently higher carriage rates of *S. aureus* and *H. influenzae* after PCV-7 implementation in children at a young age vulnerable for bacterial infections as well as in parents. In addition to the need for ongoing surveillance of nasopharyngeal bacterial carriage over time, disease surveillance is warranted to verify potential clinical consequences of these findings.

## Supporting Information

Table S1 Frequencies of nasopharyngeal carriage of individual *S. pneumoniae* serotypes in children and parents before, 3 and 4.5 years after PCV-7 implementation.
(DOCX)
