# Correction: Long-Term Effects of Pneumococcal Conjugate Vaccine on Nasopharyngeal Carriage of *S. pneumoniae, S. aureus, H. influenzae* and *M. catarrhalis*

**DOI:** 10.1371/annotation/2b5d32c3-808f-4759-8207-0a953e4ad01d

**Published:** 2012-09-27

**Authors:** Judith Spijkerman, Sabine M. P. J. Prevaes, Elske J. M. van Gils, Reinier H. Veenhoven, Jacob P. Bruin, Debby Bogaert, Alienke J. Wijmenga-Monsuur, Germie P. J. M. van den Dobbelsteen, Elisabeth A. M. Sanders

The following authors should have been designated as contributing equally to the article, and thus sharing first authorship: Judith Spijkerman and Sabine M.P.J. Prevaes. 

