# Supplementary material for: Long-Term Effects of Pneumococcal Conjugate Vaccine on Nasopharyngeal Carriage of S. pneumoniae, S. aureus, H. influenzae and M. catarrhalis
Source: PLoS One. 2012 Jun 25;7(6):e39730. doi: 10.1371/journal.pone.0039730 (PMC3382588; doi:10.1371/journal.pone.0039730)
Supplement: Table S1 — Frequencies of nasopharyngeal carriage of individual S. pneumoniae serotypes in children and parents before, 3 and 4.5 years after PCV-7 implementation. (DOCX) [file pone.0039730.s001.docx]

Table S1

Frequencies of nasopharyngeal carriage of individual *S. pneumoniae* serotypes in children and parents before, 3 and 4.5 years after PCV-7 implementation

|  | **11 months** | | | **24 months** | | | **Parents** | | |
| --- | --- | --- | --- | --- | --- | --- | --- | --- | --- |
|  | **Pre-PCV7** | **3yr Post** | **4.5yr Post** | **Pre-PCV7** | **3yr Post** | **4.5yr Post** | **Pre-PCV7** | **3yr Post** | **4.5yr Post** |
|  | no. (%) | no. (%) | no. (%) | no. (%) | no. (%) | no. (%) | no. (%) | no. (%) | no. (%) |
|  | n=319 | n=329 | n=330 | n=321 | n=330 | n=330 | n=296 | n=324 | n=326 |
| *PCV-7-serotypes:* | | | | | | | | | |
| **4** | 1 (0) | 0 (0) | 0 (0) | 1 (0) | 0 (0) | 1 (0) | 2 (1) | 0 (0) | 0 (0) |
| **6B** | 26 (8) | 12 (4)^a^ | 3 (1)^b,c^ | 43 (13) | 7 (2)^a^ | 2 (1)^b^ | 5 (2) | 0 (0)^a^ | 0 (0) |
| **9V** | 9 (3) | 1 (0)^a^ | 0 (0)^b^ | 6 (2) | 1 (0) | 0 (0)^b^ | 1 (0) | 0 (0) | 1 (0) |
| **14** | 10 (3) | 0 (0)^a^ | 1 (0)^b^ | 8 (3) | 0 (0)^a^ | 2 (1) | 6 (2) | 0 (0)^a^ | 2 (1) |
| **18C** | 6 (2) | 1 (0) | 0 (0)^b^ | 4 (1) | 2 (1) | 0 (0)^b^ | 0 (0) | 0 (0) | 2 (1) |
| **19F** | 36 (11) | 5 (2)^a^ | 4 (1)^b^ | 24 (8) | 4 (1)^a^ | 4 (1)^b^ | 9 (3) | 2 (1)^a^ | 2 (1) |
| **23F** | 34 (11) | 6 (2)^a^ | 2 (1)^b^ | 28 (9) | 0 (0)^a^ | 2 (1)^b^ | 2 (1) | 0 (0) | 0 (0) |
| *Non-PCV-7-serotypes*^d^*:* | | | | | | | | | |
| **19A** | 5 (2) | 32 (10)^a^ | 39 (12)^b^ | 9 (3) | 21 (6)^a^ | 47 (14)^b,c^ | 4 (1) | 6 (2) | 10 (3) |
| **15BC** | 7 (2) | 14 (4) | 13 (4) | 10 (3) | 14 (4) | 15 (5) | 2 (1) | 4 (1) | 4 (1) |
| **11A** | 11 (3) | 12 (4) | 13 (4) | 10 (3) | 22 (7)^a^ | 14 (4) | 3 (1) | 8 (2) | 4 (1) |
| **6C^e^** | 5 (2) | 8 (2) | 13 (4) | 5 (2) | 9 (3) | 14 (4)^b^ | 1 (0) | 1 (0) | 6 (2) |
| **21** | 1 (0) | 2 (1) | 9 (3)^b,c^ | 2 (1) | 2 (1) | 14 (4)^b,c^ | 1 (0) | 0 (0) | 1 (0) |
| **23B** | 5 (2) | 5 (2) | 7 (2) | 12 (4) | 11 (3) | 13 (4) | 0 (0) | 4 (1) | 3 (1) |
| **10A** | 1 (0) | 6 (2) | 7 (2)^b^ | 1 (0) | 8 (2)^a^ | 12 (4)^b^ | 1 (0) | 2 (1) | 2 (1) |
| **23A** | 5 (2) | 1 (0) | 7 (2)^c^ | 1 (0) | 2 (1) | 9 (3)^b,c^ | 0 (0) | 0 (0) | 4 (1) |
| **35F** | 2 (1) | 5 (2) | 2 (1) | 0 (0) | 9 (3)^a^ | 8 (2)^b^ | 0 (0) | 3 (1) | 0 (0) |
| **35B** | 0 (0) | 3 (1) | 9 (3)^b^ | 2 (1) | 2 (1) | 7 (2) | 1 (0) | 1 (0) | 3 (1) |
| **16F** | 1 (0) | 6 (2) | 7 (2)^b^ | 4 (1) | 6 (2) | 6 (2) | 3 (1) | 1 (0) | 2 (1) |
| **17F** | 1 (0) | 0 (0) | 4 (1)^c^ | 1 (0) | 3 (1) | 6 (2) | 0 (0) | 0 (0) | 0 (0) |
| **6A^e^** | 19 (6) | 11 (3) | 4 (1)^b^ | 17 (5) | 10 (3) | 4 (1)^b^ | 3 (1) | 3 (1) | 2 (1) |
| **22F** | 4 (1) | 7 (2) | 1 (0)^c^ | 2 (1) | 5 (2) | 2 (1) | 0 (0) | 2 (1) | 1 (0) |
| **NT** | 1 (0) | 6 (2) | 3 (1) | 3 (1) | 5 (2) | 4 (1) | 1 (0) | 4 (1) | 3 (1) |
| **Other** | 24 (8) | 11 (3) | 28 (8) | 18 (6) | 19 (6) | 35 (11) | 5 (2) | 10 (3) | 12 (4) |

Note. PCV-7; all serotypes included in the 7-valent conjugate vaccine. Non-PCV-7; all other serotypes not included in the 7-valent conjugate vaccine. NT; non-typeables. P-values are calculated with chi-square test or Fisher’s exact test (2-sided) where appropriate; p-value <0.05:^a^3yr Post vs. Pre-PCV-7, ^b^4.5yr Post vs. Pre-PCV-7, ^c^4.5yr Post vs. 3yr Post-PCV-7. ^d^Only non-PCV-7-serotypes with n >5 isolates in 11 or 24 month-old children or in parents are included in this table. ^e^Up to 2009, no serotype-specific antiserum was available to discriminate between serotype 6A and 6C. Therefore, all serotypes 6A found Pre-PCV-7 and 3 yr Post-PCV-7 were discriminated by PCR using primers 5106 and 1301, and primers 6C-fwd and 6C-Rev. After discrimination, only at 24 months, different serotypes were found by PCR compared to Quellung: Pre-PCV-7 3 isolates (serotypes 6B (n=1) and 14 (n=2)) and 3yr Post-PCV-7 2 isolates (serotypes 11 and 15). These serotypes were not included in this table.
